# Translational cognitive biomarkers for preclinical drug testing in neurodegenerative diseases

**DOI:** 10.1002/alz70856_103517

**Published:** 2025-12-26

**Authors:** Rodrigo Sandoval, Vladislav Novikov, Anoosha Attaran, Mohammad‐Hossein Tabatabaei, Amr Eed, Matthew Cowan, Man Ching Yau, Yasmien Abduldayem, Sahil Sharma, Wen Luo, Irina Shlaifer, Czarina Evangelista, Thomas M. Durcan, Edward A Fon, Ravi S. Menon, Gabriela Chiosis, Timothy J Bussey, Lisa M Saksida, M. Mallar Chakravarty, Vania F Prado, Marco AM Prado

**Affiliations:** ^1^ University of Western Ontario, London, ON, Canada; ^2^ The University of Western Ontario, London, ON, Canada; ^3^ Memorial Sloan Kettering Cancer Center, New York, NY, USA; ^4^ McGill University, Montreal, QC, Canada; ^5^ Montreal Neurological Institute, McGill University, Montreal, QC, Canada; ^6^ Centre for Functional and Metabolic Mapping, Robarts Research Institute, London, ON, Canada; ^7^ Memorial Sloan Kettering, New York, NY, USA; ^8^ Sloan Kettering Institute, New York, NY, USA; ^9^ Schulich School of Medicine and Dentistry, London, ON, Canada; ^10^ Robarts Research Institute, London, ON, Canada; ^11^ StoP‐AD Centre, Douglas Mental Health Institute Research Centre, Montreal, QC, Canada; ^12^ Brain and Mind Institute, London, ON, Canada

## Abstract

**Background:**

Neurodegenerative diseases such as Alzheimer's disease (AD) and Parkinson's disease (PD) are characterized by the accumulation of misfolded proteins, including amyloid‐β, tau, and alpha‐synuclein (a‐Syn). Despite extensive research, no effective disease‐modifying therapies exist. A major barrier to drug development is the poor translatability of animal models and the lack of biomarkers with predictive power for clinical efficacy. Higher‐order cognitive deficits, such as impaired reversal learning, are observed in patients with synucleinopathies, suggesting that cognitive biomarkers could be valuable in preclinical drug testing. This study evaluates whether touchscreen‐based cognitive testing enhances the predictive validity of preclinical drug testing. We assessed PU‐AD, an HSP90 epichaperome disruptor targeting the abnormal chaperone network implicated in protein misfolding disorders, and a mouse version of Cinpanemab, an anti‐a‐Syn antibody that recently failed in clinical trials, despite initial positive results in animal models.

**Method:**

Hemizygous M83 transgenic mice were intracerebrally injected with preformed fibrils (PFFs) to model synucleinopathy‐related neurodegeneration. Mice underwent cognitive assessment using the Pairwise Visual Discrimination and Reversal (PVD‐R) touchscreen task, a highly translational measure of cognitive flexibility sensitive to a‐Syn toxicity. PU‐AD and Cinpanemab were administered intraperitoneally post‐PFF injection. Drug doses matched previous animal model studies. Motor performance was assessed with grip strength, rotarod, and wire hang tests. MRI was used to detect brain atrophy. Immunohistochemical and biochemical analyses evaluated a‐Syn pathology and neuroinflammation.

**Result:**

M83/PFF‐injected mice showed significant reversal learning deficits earlier than motor deficits. Pharmacokinetic analysis confirmed PU‐AD reached brain concentrations comparable to those in other models. PU‐AD treatment rescued cognitive and motor impairments, suggesting broad neuroprotective effects. In contrast, Cinpanemab‐treated mice showed no improvements in cognition and preliminary data suggest no improvements in motor symptoms. Ongoing experiments are evaluating pathology and brain atrophy.

**Conclusion:**

The lack of Cinpanemab efficacy in our pre‐clinical testing pipeline aligns with clinical trial results. The effectiveness of PU‐AD in reducing both cognitive and motor impairments suggests it may have broad therapeutic potential in synucleinopathies. We suggest that touchscreen‐based cognitive testing enhances the predictive validity of preclinical drug evaluation.

